# Homœopathy

**Published:** 1880-09

**Authors:** 


### BIBLIOGRAPHICAL.

*Homœopathy: What is it?* By A. B. Palmer, A. M., M. D. Professor of Pathology, &c., University of Michigan, Detroit. Geo. S. Davis, Publisher.

In the introduction to this little book of 100 pages, Dr. Palmer states that the greater part of the matter it contains was given in lectures before the Medical Class of the University of Michigan, doubtless in the war that resulted from the introduction of Homœopathy into that school by legislative action. Whether written under such stimulus or not, it is certainly a most searching inquiry into the fallacies and pretentions of the doctrine of Hahnemannic theories and practice. It is hard to see how a stronger case could be made of utter pretension and humbuggery on the part of a profession than is here presented. The arraignment of *similia similibus*, its folly and weakness, is strong; the utter absurdity of decillionth dilutions; the fact that its professed advocates secretly abandon it, while claiming to follow it; the folly of symptomatic treatment of disease, as practiced by the Homœopaths; all this and much more that is interesting, is here presented in a strong and even ludicious light.

It is indeed difficult to believe that Hahnemann really had faith in his own dogmas, although announcing them with such boldness and confidence. But we must remember that he was at one time a believer in Mesmerism, and had at one time charge of an insane asylum. Possibly in this last fact, on the theory that insanity, like measles is catching, may be found the solution of Homœapathp. No other hypothesis, it would seem, could explain the etherealism of the following extract from the "Lesser writings of Hahnemann," in which he is explaining the wonderful results of "shaking" the tinctures. He writes:

"This result, [of potentization] so incomprehensible to the man of figures, goes so far that we must set bounds to the succussion process, in order that the degree of attenuation be not overbalanced by the increased potency of the medicine, and in that way the highest attenuations become too active. If we wish for example to attenuate a drop of the juice of *Sundew* to the thirtieth degree, but shake each of the bottles with twenty or more succussions from a powerful arm, in the hand of which the bottle is held, in that case this medicine, which I have discovered to be the specific remedy for that frightful epidemic, whooping-cough of children, will have become so powerful in the fifteenth attenuation (spiritualized) that a drop of it given in a tea-spoonful of water, would endanger the life of such a child; whereas, if each dilution bottle was shaken but *twice* (with two strokes of the arm) and performed in this manner up to the thirtieth attenuation, a sugar globule, the size of a poppy-seed moistened with the last attenuation, cures this terrible disease with this single dose," etc.

Hahnemann it seems was "cute" if "cracked," as his assertion that "wine and alcohol are not *potentized* by their dilutions," proves. He knew better than to fly in the face of drinkers of his day.

Dr. Palmer shows that even in the milk-sugar used as the vehicle of administration of these so-called remedies, there is more lime, potash, soda, etc. than in the tinctures used. These, then, would be as efficient, when these materials were indicated as if unindicated. Of course it is idle to argue with such men, when they assert, as Hahnemann, or rather Crosiero, one of his disciples, that his wife was cured of a violent pleurisy in five hours by only smelling a globule moistened with the thirtieth dilution of something, which it seems retained all its powers for eighteen or twenty years after the moistening although exposed to sun and air. And the quintillionth attenuation of a grain of gold leaf will prevent suicide. That the lack of money does drive many to self-destruction is evident from the daily reports in the newspapers; but that so small a quantity of gold as this, sniffed up the nostrils will prevent all this, is a cheap method of prevention of suicide which should be more generally known; though it may be that some peculiarity in the preparation may be needful. Is the trituration from *a gold filling in a tooth* sufficient? But we cannot follow Dr. Palmer. Suffice it that this book is a most amusing one ; not that the Dr. tries especially to make it so, but by the reproduction of the absurdities of the originators and followers of homæopathy. Any one who wishes a good hearty laugh will surely find cause for it here ; and any Allopath who wants a condusive argument to put in the hands of a patient inclined to "little pills," will do well to loan him or her this book. H.

